# Improving Minutiae Image of Latent Fingerprint Detection on Non-Porous Surface Materials under UV Light Using Sulfur Doped Carbon Quantum Dots from Magnolia Grandiflora Flower

**DOI:** 10.3390/nano12193277

**Published:** 2022-09-21

**Authors:** David Nugroho, Won-Chun Oh, Saksit Chanthai, Rachadaporn Benchawattananon

**Affiliations:** 1 Department of Integrated Science, Faculty of Science, Khon Kaen University, Khon Kaen 40002, Thailand; 2 Department of Advanced Materials Science and Engineering, Hanseo University, Seosan-si 356-706, Korea; 3 Materials Chemistry Research Center, Department Chemistry and Center of Excellence for Innovation Chemistry, Faculty of Science, Khon Kaen University, Khon Kaen 40002, Thailand

**Keywords:** minutiae pattern, carbon dots, latent fingerprint, nanoparticle, forensic science

## Abstract

In this study, carbon quantum dots (CQDs) from Magnolia Grandiflora flower as a carbon precursor were obtained using a hydrothermal method under the optimized conditions affected by various heating times (14, 16, 18, and 20 min) and various electric power inputs (900–1400 W). Then, hydrogen sulfide (H_2_S) was added to dope the CQDs under the same manner. The aqueous solution of the S-CQDs were characterized by FTIR, XPS, EDX/SEM, and TEM, with nanoparticle size at around 4 nm. Then, the as-prepared S-CQDs were successfully applied with fine corn starch for detection of minutiae latent fingerprints on non-porous surface materials. It is demonstrated that the minutiae pattern is more clearly seen under commercial UV lamps with a bright blue fluorescence intensity. Therefore, this research has proved that the S-CQDs derived from plant material have a better potential as fluorescent probes for latent fingerprint detection.

## 1. Introduction

Fingerprints are considered to be a gold standard for identifying biometrics and scientific evidence in the police field. Fingerprints are unique, and because each person has a different pattern of minutiae, they cannot be changed, unless someone gets into an accident that causes them to not have minutiae latent fingerprint, and they are easy to verify based on the capillary tube-shaped channels at the fingertips, which are connected to the sweat glands [1,2,3,4,5,6,7,8]. Fingerprint detection is currently carried out using some physical or chemical processes to detect the residue of latent fingerprint amino acids, oils, and others. This is because the characteristics of latent fingerprints cannot be seen directly by the naked eye [9,10,11,12,13,14].

Carbon dots (CQDs) are a member of the nanomaterial family, with the characteristics of a nanometer particle size, biocompatibility, low toxicity, and water-solubility. These CQDs have a photoluminescence characteristic that can be used in several applications, such as bioimaging, as drug carriers, for gene delivery, metal ion detection, sensing, and as nanothermometers. There are two methods for CQDs synthesis. The first is a top-down method by cutting the carbon source material, and it makes use of a bottom-up method involving the carbonization of small molecules. The hydrothermal and microwave method is the second method, which is a bottom-up approach possessing the advantages of energy efficiency, low cost, and convenient operation, and it is mainly used to carbonize organic matter to form luminescence at high temperatures and pressures [15,16,17]. In this study, we prepared an S-doped-CQDs nanocomposite material synthesized using microwave methods in various conditions to obtain the optimum product.

## 2. Methods and Matrial

### 2.1. Chemicals and Reagents

Hydrogen sulfide was from Kanto Chemical Co. Inc, Tokyo, Japan. Magnolia Grandiflora flower was collected during a short spring season from our Hanseo University flower park at the Department of Advance Materials Science and Engineering, Haemi-myeon, Seosan-si, Korea.

### 2.2. Synthesis

#### 2.2.1. Preparation of Magnolia Grandiflora Powder

Magnolia Grandiflora flower was cut to small sizes and dried for 48 h at 75 °C in an oven, ground using a laboratory mortar, and then kept at room temperature for further use.

#### 2.2.2. Preparation of S-CQDs with Microwave Method

Here, 5 g of powder was dispersed in 250 mL of DI water, then stirred for 30 min at room temperature. Synthesis was conducted using a commercial microwave at various times (14, 16, 18, and 20 min) and varying wattages (900, 1000, 1100, 1200, 1300, and 1400 W). After obtaining the optimum condition for synthesizing S-CQDs. 5 g of flower powder and 0.05 g of H_2_S added was synthesized in the optimum condition (1400 W and 20 min), and after synthesis, it was centrifuged at 5000 rpm for 10 min and filtered using Whatman paper, and the final product of the S-CQDs (a brown solution) was dried in an oven at 65 °C for 24 h to make powder. Lastly, the final powder of the S-CQDs was stored at room temperature.

#### 2.2.3. Preparation of S-CQDs Detecting Powder

To begin, 0.2 g of S-CQDs and 5 g of commercial starch powder were mixed with 20 mL of DI water. Then, the solution was stirred at RT (room temperature) for 12 h, and then dried at 65 °C for 24 h to make it become powder in an oven. The powder of S-CQDs detection powder was then stored at room temperature.

## 3. Results and Discussion

### 3.1. Fourier-Transform Infrared Spectroscopy (FTIR)

**Figure 1:**
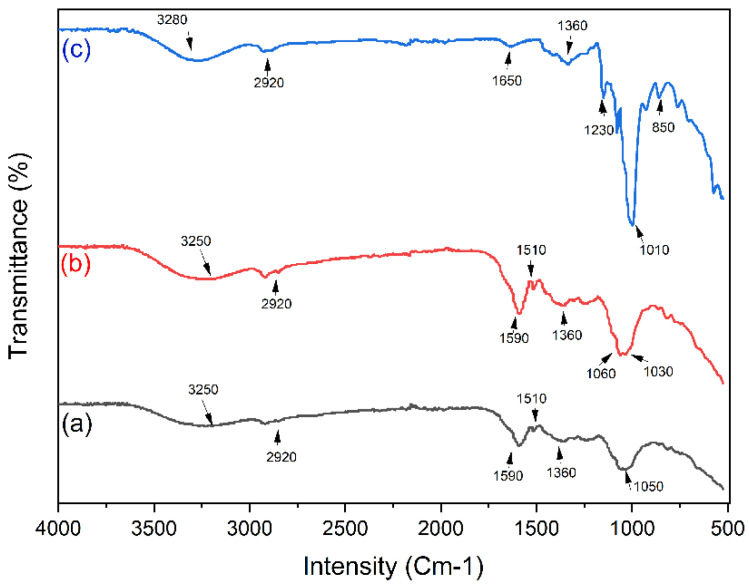
FTIR of (a) CQDs, (b) S-CQDs, and (c) S-CQDs/starch.

Functional groups from Magnolia Grandiflora were analyzed by FTIR as shown in Figure 1(a), which shows the O-H bond at 3250 cm^−1^. At 2920 cm^−1^ a C-H stretching alkene bond was found, ester groups (C=O stretching) were found between 1590 cm^−1^, a N–H bending vibration bond was found at 1510 cm^−1^, ester groups (C=O stretching) were found at 1360cm^−1^, and the C-O-C bond was found at 1050 cm^−1^. Figure 1(b) shows where the stretching vibration of O=S=O can be seen at 1030 cm^−1^, the O-H stretching bond at 3250 cm^−1^, the alkene bond (C-H stretching) at 2920 cm^−1^, the C=O stretching of ester groups at 1590 cm^−1^, N–H bending vibration bond at 1510 cm^−1^, C=O stretching of ester groups at 1360 cm^−1^, and the C-O-C bond at 1060 cm^−1^. Figure 1(c) shows the results of FTIR analysis from CQDs with corn starch, where it can be seen that the O-H stretching bond appears at about 3280 cm^−1^, the alkane bond of the C-H bond at 2920 cm^−1^, -OH vibration at 1360 cm^−1^, C-O vibration at 1230 cm^−1^, C-O-C stretching bond at 1010 cm^−1^, and that C=C bonding trisubstituted at 850 cm^−1^ [18,19,20,21,22,23,24,25,26,27,28,29,30,31].

### 3.2. X-ray Diffraction Analysis (XRD)

**Figure 2:**
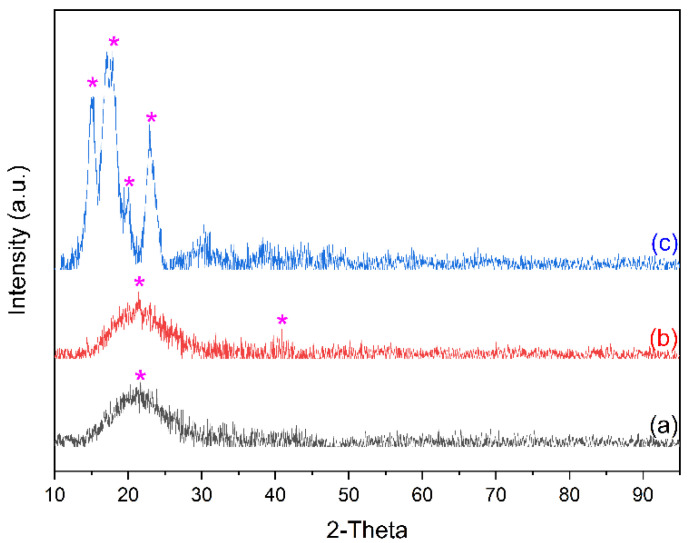
XRD of (a) CQDs, (b) S-CQDs, and (c) S-CQDs/starch.

X-ray diffraction (XRD) analysis revealed the crystallinity and phase of the material on the synthesized CQDs. Figure 2(a) shows that the diffraction peaks were located at 2θ = 21.38° (002) with an interplanar spacing of 0.415 nm/4.15 and peaks at 26.38° (002) with an interplanar spacing of 0.33 nm/3.34 Å, which correspond to the diffraction line of carbon. Figure 2(b) shows that the diffraction peaks were located at 2θ = 21.38° (002) with an interplanar spacing of 0.415 nm/4.15 Å and peaks at 26.38° (002) with an interplanar spacing of 0.33 nm/3.34 Å, which correspond to the diffraction line of carbon, and they show diffraction peaks at 2θ = 40.9° (113) with an interplanar spacing of 0.23 nm/2.28 Å. After being mixed with starch powder, as shown in Figure 2(c), the crystal structure of S-doped-CQDs shifted due to the combination of carbon dots with flour; the flour had the characteristic of a powder that was lumpier, and its diffraction peaks were located at 2θ = 11.67° (001), 18.84° (111), 20.38° (020), and 24.77° (211) [32,33,34,35].

### 3.3. Elemental X-ray Photoelectron Spectroscopy (XPS) Analysis 

**Figure 3:**
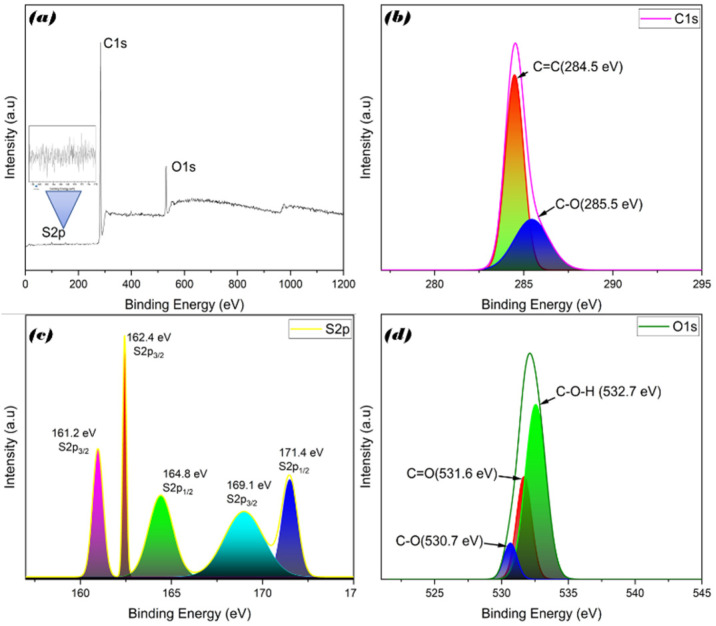
XPS of (**a**) survey spectrum of S-CQDs, (**b**) C1s, (**c**) S2p, and (**d**) O1s peaks.

The elemental carbon bonding, oxygen bonding, compositions, and sulfur bonding of S-doped-CQDs were analyzed using XPS. The spectrum of the S-doped-CQDs is shown in Figure 3a. Strong peaks at 283.0 and 530.0 eV may, respectively, indicate the energy binding carbon (C 1s) and oxygen (O 1s). The XPS spectra of C 1s (Figure 3b) indicates inadequate carbonization during the heating process by using the microwave method of Magnolia Grandiflora, which reveals the presence of C–O and C=C functional groups with deconvoluted binding energies of 284.5 and 285.5 eV, respectively. Substantial numbers of oxygen functional groups were detected in the high-resolution XPS spectra of S-doped-CQDs (Figure 3d) at 531.6, 530.7, and 532.7 eV, which, respectively, corresponded to the C=O, C–O, and C-O-H bonds. The S 2p spectra of S-doped-CQDs (Figure 3c) has two peaks centered at 161.2 and 171.4 eV, indicating that S exists in two forms. The former peak may be deconvoluted into three different components at 161.2, 162.4, and 169 eV, which agree with the S2p_3/2_, while the other two peak can be deconvoluted into components at 164.8 and 171.4 eV, which agree with S2p_1/2_ [36,37].

### 3.4. Morphological and Optical Property Analysis

**Figure 4:**
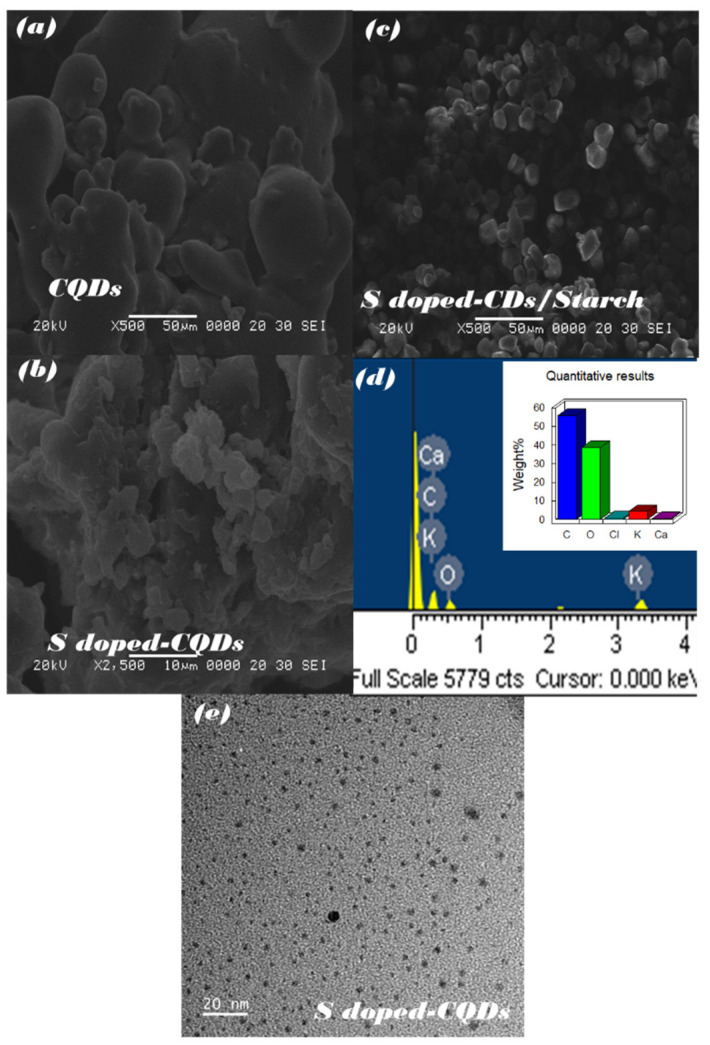
FIB-SEM of (**a**) CQDs, (**b**) S-CQDs, and (**c**) S-CQDs/starch; EDX of (**d**) S-CQDs; TEM of (**e**)-CQDs.

Figure 4 shows an image analysis by a TEM, which is consistent with the focused ion FIB-SEM image. The S-doped-CQDs and CQDs has been analyzed for the characterization using TEM, and it is shown in Figure 4e it was found that the particle size of S-doped-CQDs was around 4 nm. Under the FIB-SEM, the surfaces of the CQDs and S-doped-CQDs have been analyzed, and it can be seen in Figure 4a that the surface of CQDs has a smooth texture and a circle shape. Figure 4b shows that, when CQDs mixed with H_2_S become S-doped-CQDs, the texture characteristic changes from smooth to a rough and uneven texture. After the S-doped-CQDs are mixed with starch, it can be seen in Figure 4c that they combine perfectly and make a particle with a sharp texture. The energy-dispersive X-ray (EDX) of S-doped-CQDs has been analyzed, and it can be seen in Figure 4d that the major elements are C (55.92%) and O (36.57%).

**Figure 5:**
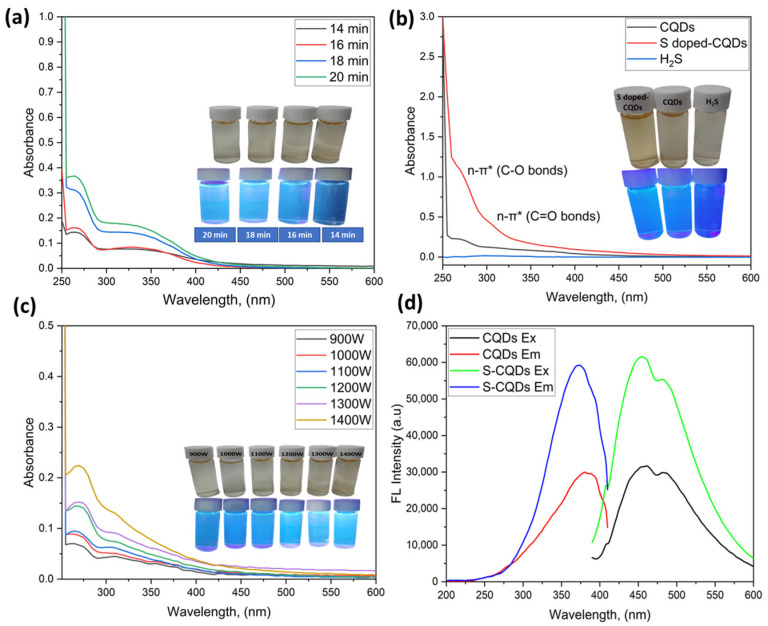
Synthesis optimization of S-CQDs: (**a**) Uv–Vis of the effect of heating time, (**b**) Uv–Vis of the effect of H_2_S on absorption of CQDs, (**c**) Uv–Vis of the effect of hydrothermal treatment (W), (**d**) FL Spectrophotometer of optimum condition (S/W:5/5).

Optical properties were analyzed in terms of UV–visible absorption and a commercial UV lamp (365 nm). By following Atchudan et al., the CQD was slightly modified by various synthesis durations using microwave methods in varying conditions of time, namely 14, 16, 18, and 20 min, and it was shown that 20 min leads to more blue light under UV lamp, and that 20 min is the optimum condition under UV–Vis spectrophotometer (Figure 5a). The optimum condition for CQDs synthesis has been obtained under microwave by using varying wattage conditions, namely 900, 1000, 1100, 1200, 1300, and 1400 W. Further, under a UV–Vis spectrophotometer, it can be seen that the optimum condition is 1400 W and that there is more blue light under the UV lamp (Figure 5c). After obtaining the optimum conditions, the CQDs were doped with H_2_S to become S-CQDs by adding 0.05 g H_2_S in the optimum condition of the CQDs. The optical properties were monitored, and it was seen that H_2_S can enhance the absorbance from CQDs, and that under UV lamp it also shows more blue light. Figure 5b shows the UV–Vis absorption of CQDs, where it can be seen that S-CQD displays an absorption peak centered at 270 nm, which is assigned to the n→π* transition of C=O [38] and, for S-CQDs, there is an absorption peak at 310 nm that is assigned to the n→π* transition of C-O [39]. This is similar to previously reported results for carbon quantum dot-based materials, and we can also see that S-CQDs have higher band gap energies than others. After obtaining the optimum condition from the Uv–Vis spectrophotometer analysis, the optimum condition has been checked using a fluorescence spectrophotometer. Figure 5d show the comparison between CQDs and S-CQDs. Here, S-doping from H_2_S has the effect of increasing the intensity of CQDs, with the optimal condition excitation of 370 nm and emission of 455 nm.

### 3.5. Detection of Latent Fingerprints

#### 3.5.1. Comparison of S-CQDs and CQDs for Latent Fingerprint Detection on Non-Porous Material Surface

The detection of latent fingerprints was tested with the aid of a volunteer who printed his index finger on non-porous material surfaces. Detection powder was brushed on the non-porous surface material, and the latent fingerprint was detected under a normal lamp (LED lamp) and UV lamp (wavelength 365 nm). Latent fingerprint detection was tested on many type of non-porous material surfaces, such as glass, iron, and aluminum foil. Table 1 shows that the detection powder could detect the latent fingerprints on many types of non-porous surface material. Indeed, S-CQDs have a clearer and brighter result under UV lamp, and they can show the minutiae of latent fingerprints compared to the CQDs, but in the normal commercial lamp, neither of them showed it clearly, due to the color of the powder.

#### 3.5.2. Effect of Particle Size Powder for Latent Fingerprint Detection

Capillary lipid protrusions on the tips of the fingers, which contain rows of pores connected to the sweat glands, ensure that everyone has different patterns of fingerprints, and there are very close distances between the lines. In fingerprint detection, particle size dramatically affects the final result from the detection of latent fingerprints [40]. Therefore, in this research, the latent fingerprints have been checked under varying particle sizes. After the S-CQDs detection powder was prepared, the detection powder was sieved using a testing sieve with various pore sizes of 38 μm, 53 μm, and 73 μm. The S-CQD detection powder was brushed onto the material surfaced, and it was detected under a normal lamp and a UV lamp. The result shows that the smallest (38 μm) particles can show the latent fingerprint minutiae in the most detail (red = bifurcation; yellow = termination; green = island; orange = lake), and this will help criminal investigations (Table 2).

#### 3.5.3. Comparison of S-CQDs and Commercial Powder on Latent Fingerprint Detection on Non-Porous Material Surfaces

The detection of latent fingerprints was aided by a volunteer who printed his index finger on non-porous material surfaces. Detection powder was brushed on the fingerprint, which was then detected under a normal lamp (LED lamp) and a UV lamp (wavelength 365 nm). Table 3 shows the comparison of the detection of latent fingerprints on a glass surface material. The S-doped-CQDs have a clearer result under UV lamp; of the total minutiae, nine can be detected, but in the commercial powder it appears clearer under the normal lamp, with a total of eight minutiae detected (red = bifurcation; yellow = termination; green = island; orange = lake). 

#### 3.5.4. Temperature and Storage Time and for Latent Fingerprint Detection

The effects of storage time and temperature for detecting latent fingerprints have also been tested in this research. In this testing, we used a glass material, which had been kept at different temperature conditions (−10 °C, Room temp Advanced Materials Science and Engineering Lab, Hanseo University, Korea., and 60 °C) at various times. Moreover, the result can be seen in Table 4, which shows that the detection power of S-doped-CQDs can be used to clearly detect the minutiae of latent fingerprints at room temperature until 120 h.

## 4. Conclusions

In this work, we synthesized novel fluorescent S-CQDs via a microwave method by using Magnolia grandiflora and hydrogen sulfide as sulfur source. The as-prepared S-CQDs exhibited excellent photostability under various conditions, and the S-CQDs have been characterized under TEM, SEM, XRD, EDX, FTIR, and UV–Vis. Furthermore, S-CQDs are very suitable for the detection of latent fingerprints on non-porous surface materials, and they can also be applied in cases of varying temperatures and varying times of storage. This study provides the detection of latent fingerprint on non-porous material surfaces, and it shows promising results for forensic sciences.

## Figures and Tables

**Table 1:** Detection on non-porous surface material using S-CQDs/starch vs. CQDs/starch under normal and UV lamps.

Material Type	CQDs/Starch (Normal Lamp)	CQDs/Starch (UV Lamp)	S-CQDs/Starch (Normal Lamp)	S-CQDs/Starch (UV Lamp)
Knife surface	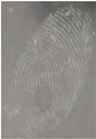	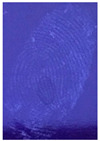	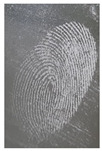	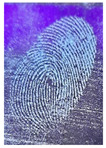
Glass surface	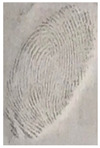	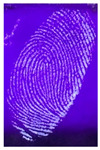	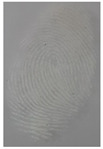	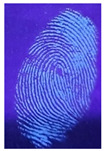
Aluminum surface	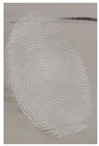	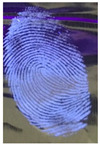	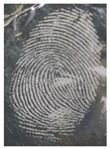	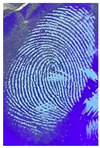

**Table 2:** Detection of latent fingerprints using different particle sizes of S-CQDs/corn starch under normal and UV lamps.

Particle Size (μm)	S-CQDs/Corn Starch (Normal Lamp)	S-CQDs/Corn Starch (UV Lamp)	Total Minutiae of Latent Fingerprint Can Be Detect
38	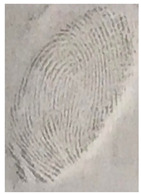	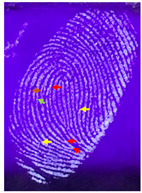	Bifurcation: 3 Minutiae Termination: 2 Minutiae Island: 1 Minutiae Orange: 1 Minutiae Total: 7 Minutiae
53	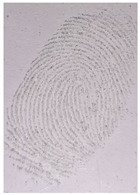	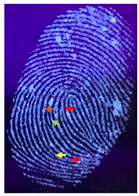	Bifurcation: 2 Minutiae Termination: 1 Minutiae Island: 1 Minutiae Orange: 1 Minutiae Total: 5 Minutiae
73	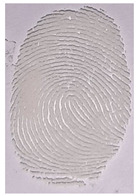	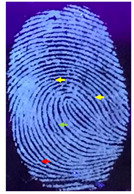	Bifurcation: 1 Minutiae Termination: 2 Minutiae Island: 1 Minutiae Total: 4 Minutiae

**Table 3:** Detection of latent fingerprints on a glass surface using S-CQDs/starch vs. commercial powder under normal and UV lamps.

Material Type	Commercial Powder (Normal Lamp)	Commercial Powder (UV Lamp)	S-CQDs/Starch (Normal Lamp)	S-CQDs/Starch (UV Lamp)
Glass	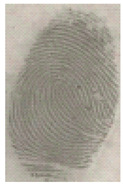	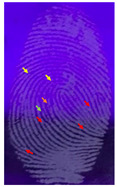	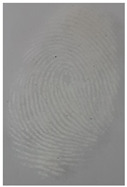	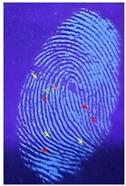

**Table 4:** The effect of storage time and temperature for latent fingerprints detection.

XT	−10 °C	Room Temp. (25–27 °C)	60 °C
24	MD	MD	MD
48	LFD	MD	MD
72	LFD	MD	MD
96	LFD	MD	LFD
120	LFD	MD	LFD

Abbreviations are as follows: MD, minutiae detected; LFD, latent fingerprint detected.

## Data Availability

Data can be available upon request from the authors.

## Sample Availability

Samples are available from the authors.
